# Putting the data before the algorithm in big data addressing personalized healthcare

**DOI:** 10.1038/s41746-019-0157-2

**Published:** 2019-08-19

**Authors:** Eli M. Cahan, Tina Hernandez-Boussard, Sonoo Thadaney-Israni, Daniel L. Rubin

**Affiliations:** 1 New York University School of Medicine, New York, NY USA; 2 Department of Pediatric Orthopaedics, Stanford University, Palo Alto, CA USA; 3 Department of Biomedical Data Sciences, Stanford University, Palo Alto, CA USA; 4 Department of Medicine, Stanford University, Palo Alto, CA USA; 5 Department of Surgery, Stanford University, Palo Alto, CA USA; 6 Department of Radiology, Stanford University, Palo Alto, CA USA

**Keywords:** Machine learning, Medical ethics, Public health, Quality control, Data integration

## Abstract

Technologies leveraging big data, including predictive algorithms and machine learning, are playing an increasingly important role in the delivery of healthcare. However, evidence indicates that such algorithms have the potential to worsen disparities currently intrinsic to the contemporary healthcare system, including racial biases. Blame for these deficiencies has often been placed on the algorithm—but the underlying training data bears greater responsibility for these errors, as biased outputs are inexorably produced by biased inputs. The utility, equity, and generalizability of predictive models depend on population-representative training data with robust feature sets. So while the conventional paradigm of big data is deductive in nature—clinical decision support—a future model harnesses the potential of big data for inductive reasoning. This may be conceptualized as clinical decision questioning, intended to liberate the human predictive process from preconceived lenses in data solicitation and/or interpretation. Efficacy, representativeness and generalizability are all heightened in this schema. Thus, the possible risks of biased big data arising from the inputs themselves must be acknowledged and addressed. Awareness of data deficiencies, structures for data inclusiveness, strategies for data sanitation, and mechanisms for data correction can help realize the potential of big data for a personalized medicine era. Applied deliberately, these considerations could help mitigate risks of perpetuation of health inequity amidst widespread adoption of novel applications of big data.

## Past: dichotomy between the data and the algorithm

The tsunami of big data—harnessed most prominently through predictive algorithms and machine learning—has swept across healthcare in recent years.^1^ Demonstrated applications exist for many discrete clinical scenarios (Table 1). Applications have also enveloped biomedical research, health systems utilization review, and medical curricular redesign.^2–4^

**Table 1:** Selected current machine learning applications using big data in healthcare

Specialty	Clinical Problem	Methodology	Source
Radiology	Coronary artery calcification Thoracic lesion inspection Mammography	Enhanced image reconstruction Improved feature detection (diagnostic) Improved feature interpretation (prognostic)	Giger ML. J Am Coll Radiol. 2018;15(3 Pt B):512–20.
Pathology	Breast cancer	Enhanced image reconstruction Improved feature detection (diagnostic) Improved feature interpretation (prognostic)	Beck AH, Sangoi AR, Leung S, Marinelli RJ, Nielsen TO, van de Vijver MJ, et al. Sci Transl Med. 2011;3(108):108ra13.
Ophthalmology	Diabetic retinopathy	Enhanced image reconstruction Improved feature detection (diagnostic)	Gulshan V, Peng L, Coram M, Stumpe MC, Wu D, Narayanaswamy A, et al. JAMA. 2016;316(22):2402–10.
Emergency Medicine	Clinical triage	Use of retrospective EHR data for training→outcome prediction upon new patient presentation	Hong WS, Haimovich AD, Taylor RA. PLoS One. 2018;13(7):e0201016.
Cardiology	Heart failure outcomes	Use of retrospective EHR data for training→outcome prediction upon new patient presentation	Ahmad T, Lund LH, Rao P, Ghosh R, Warier P, Vaccaro B, et al. J Am Heart Assoc. 2018;7(8).
Neurology	Ischemic stroke outcomes	Use of retrospective EHR data for training→outcome prediction upon new patient presentation	Asadi H, Dowling R, Yan B, Mitchell P. PLoS One. 2014;9(2):e88225.
Dermatology	Melanoma staging	Enhanced image reconstruction Improved feature detection (diagnostic)	Gautam D, Ahmed M, Meena YK, Ul Haq A. Int J Numer Method Biomed Eng. 2018;34(5):e2953.

However, such algorithms—agnostic to the sources, or validity, of the big data used for training—have the potential to worsen preexisting demographic disparities in healthcare.^5^ Racial biases anchored in historically biased training datasets have led to racially biased predictive models for criminal justice, hiring decisions, allocation of social services/benefits, issuance of supportive housing, and evaluation of child abuse.^6,7^ In biomedicine, algorithms have also exhibited racial biases: for example, in prognostic models designed from the Framingham Heart Study, and precision medicine protocols based predominantly on European ancestral genotypes.^8–10^

The algorithms are often blamed for these deficiencies.^11^ However, we assert that the data used to train these algorithms bears greater responsibility. The concept of “garbage in, garbage out” is of the utmost importance for medical algorithms trained on healthcare datasets and impacting patients downstream.^12^

In this paper we (1) argue that existing big datasets are frequently limited in their inclusiveness—an issue potentially magnified by digitized devices in the future (2) examine how, if these datasets are leveraged by algorithms in an uncorrected manner, they may lack representativeness, and thus could potentially exacerbate health disparities (3) provide recommendations to improve the usefulness of future datasets, to deliver on big data’s potential for facilitating personalized healthcare.

## Present: confluence between the data and the algorithm

Deficiencies in the data inexorably compromise the algorithm. The algorithm is the terminal node in the big data value-chain: the generation, sanitization, transmission, and storage of data all precede its final predictions.^13^ The integrity of unbiased, clinically useful data depends upon the reliability of sources such as electronic health record notes and remote sensors. Its transmission relies upon the fidelity of decentralized software. Its storage depends upon the security of local and cloud-based servers.^13^ In this way, big data does not refer to headline-grabbing algorithms producing statistically significant outputs in isolation. Rather, those outputs should be viewed as inevitable byproducts of preceding inputs.

Big data has been defined by “4 V’s”: volume, velocity, variety, and veracity. While the latter two promote replicability, the volume and velocity of data have been leveraged more routinely to date.^14^ Development of algorithms has focused on the collection of data—and more data. Investigators and inventors clamor for data, focusing on its quantity rather than its quality. For example, a recent review identified 15 devices developed in recent years for continuous electrocardiographic (ECG) monitoring, yet only a minority of these performed any appraisal of the quality or usefulness of these vast accumulated data.^15^ Nevertheless, the virtue of algorithms mobilizing big data has seemed ironclad by this immensity of *N* value.

Yet, data are not necessarily useful simply because they are voluminous. The abundance of data cannot presuppose its needed diversity, representative of the populations the algorithms seek to serve. Rather, the multiplicity of data collection media, mechanisms, and contexts may produce additional susceptibility to compromising biases.^16^ This is especially true for data derived from informal sources (such as smartphones and connected wearable devices), which are not subject to methodological, hypothesis-driven rigor characterizing classical scientific data collection.^17^ Fitbit accelerometer data have shown considerable differences across step counts, intensity scores, and calculated metabolic rates from research-grade instruments, for example.^18^ As highlighted by Zhang et al., “an important concept of big data is that assembly of the data is not on purpose”.^19^

Two well-defined forms of bias warrant additional discussion: sampling bias and observation bias. Sampling bias—whereby certain patient cohorts are absent from the inputs—yields nonrepresentative algorithmic outputs. Currently, significant disparities exist in the patterns of smartphone, mobile sensor, and other device use, such that the pipelines of big healthcare data are homogenous and lack demographic diversity.^20^ The very populations who might benefit most from optimized medical interventions—including the poor, the elderly, the rural, and the disabled—are among the least likely to be using platforms generating big data.^21^ In addition, consent necessary for capturing data may be withheld in marginalized populations whose historical mistreatment by biomedicine has led to a lack of faith in and use of healthcare systems (such as African-Americans following Tuskegee).^22^ This digital redlining prompts algorithmic outputs that have inconsistent utility across populations. For example, it leads to misweighting of cardiovascular risk factors between populations, leading to phenomena such as understatement of HDL risk in Hispanic populations and of diabetes risk in African-American populations.^8^

The second form of bias is observation bias, denoting the systematic miscalibration of measurement. Measurement error has been observed in connected devices across a variety of healthcare specialties—such as in sphygmomanometry used for blood pressure assessment^23^ (Table 2). Yet while miscalibration is correctable, in a review of the 2016 high-impact literature (defined in terms of publication in one of the top 12 biomedical journals), fewer than half of all articles considered observation bias, and only 7% corrected for it.^24^ Introduction of measurement uncertainty to studies that did not consider it compromises initial findings, as demonstrated in simulations using blood pressure to predict cardiovascular disease.^25^

**Table 2:** Documented instances of measurement error using connected devices

Specialty	Clinical problem	Device/Instrument	Source
Rehabilitation medicine	Ambulation exercise tolerance	Accelerometer	Yang Y, Schumann M, Le S, Cheng S. PeerJ. 2018;6:e5775.
Orthopedics	Range of motion	Digitized protractor/goniometer	Awatani T, Enoki T, Morikita I. J Phys Ther Sci. 2017;29(10):1869–73.
Occupational health	Pneumoconioses	Environmental monitor	de Nazelle A, Seto E, Donaire-Gonzalez D, Mendez M, Matamala J, Nieuwenhuijsen MJ, et al. Environ Pollut. 2013;176:92–9.
Cardiology	Hypertension ischemic heart disease	Smartphone sphygmomanometer	Lee ES, Lee JS, Joo MC, Kim JH, Noh SE. Ann Rehabil Med. 2017;41(1):129–37.
Infection disease	Microbial outbreaks	Crowdsensors	Edoh T. J Med Syst. 2018;42(5):91.
Neurology	Gait abnormality Parkinson’s disease	Smartphone gyroscope	Ellis RJ, Ng YS, Zhu S, Tan DM, Anderson B, Schlaug G, et al. PLoS One. 2015;10(10):e0141694.
Otolaryngology	Hearing loss	Ambient sonography	Ventura R, Mallet V, Issarny V, Raverdy PG, Rebhi F. J Acoust Soc Am. 2017;142(5):3084.
Endocrinology	Prediabetes diabetes	Glucometer	Vettoretti M, Facchinetti A, Sparacino G, Cobelli C. Conf Proc IEEE Eng Med Biol Soc. 2015;2015:2359–62.
Opthalmology	Physical examination	Optical biometer	Rozema JJ, Wouters K, Mathysen DG, Tassignon MJ. Am J Ophthalmol. 2014;158(6):1111–20 e1.

As stated by Chiolero, “big data” do not speak by themselves any more than “small data”.^26^ Acceptance of the veracity of data inputs on account of volume overlooks the hazardous underbelly of volume, in its ability to amplify falsity. Even for big data, “nothing is too big to fail”.^26^

## Future: interdependence between the data and the algorithm

Occult flaws in the data used to train algorithms bear implications both on the predictions that are generated by the data (amplifying false positives), and those that are not (compounding false negatives).

### False negatives: valid predictions missed by the algorithm due to flaws in the data

The generalizability of models depends on representative training datasets. In both structured and unstructured models, representativeness necessitates a large feature set reflective of diversity in the broader population.

Yet, existing clinical data often lacks diverse subgroups (as discussed via sampling bias).^20,21^ Amplifying this issue, per one review, the majority of algorithms based on EHRs failed to correct for any missing data, and fewer than 10% corrected for all missing dimensions.^27^

Furthermore, introduction of heterogeneity relies on the conceptualization of heterogeneity—which is to say, the cognitive feature set appreciated by research investigators. Narrowness of problem representation from a methodologic standpoint leads to narrowness of algorithmic problem-solving capability. As Loscalzo and Barabasi describe, a reductive “Oslerian tradition of [linear and mechanistic] clinicopathological correlation” in disease conceptualization begets a constricted set of simplified disease profiles.^4^ For example, the poor predictive power of findings from genomic studies has been shown to arise from exclusion of environmental factors—which can contribute as much towards phenotypic variation as do genetic traits.^28^ These problems may be intensified if reductive feature sets are explicitly programmed into the models. Chen and Asch have observed that “no amount of algorithmic finesse or computing power can squeeze out information that is not present”.^17^

### False positives: invalid predictions made by the algorithm due to flaws in the data

The compromise of predictions that are generated by data is potentially more insidious. Often these arise from unconsidered confounders. Nurtured on data that exists but has minimal veracity, algorithms will in turn make minimally generalizable predictions.^29^

Important causes beyond observation bias in healthcare include changes in medical coding practices or variations in clinical practice, both of which form the very basis of clinically derived datasets.^19,30^ This may lead to (i) overfitting, in which predictions are internally valid, but externally invalid (the computational equivalent of hubris) or (ii) noncausal associations, which are both internally and externally valid, but not clinically impactful.^31^

The greater danger of noncausal associations is their potential to perpetuate incorrect assumptions. This includes those related to race and socioeconomic based health disparities.^6,7^ For example, black infant and maternal mortality are more than two and four times those of their white counterparts, yet this association is unlikely to be solely biologic. A total of 95% of genetic differences occur within races rather than between them, and only 14% of these exhibit clinically relevant effects.^32^ Causative factors for these associations cannot be parsed by datasets containing race categories alone (and lacking more granular psycho-social considerations).

In short, algorithms trained on biased, uncorrected datasets are vulnerable to exacerbation of false negatives and false positives alike. Flawed predictions in silico misguide clinical practice and may harm patients when translated in vivo.^29^ For example, in one study unadjusted Framingham risk scores demonstrated underestimation of cardiovascular mortality by up to 48% in diverse demographic groups and led to undertreatment of 29% of the cohort.^33^

## The old paradigm: deductive reasoning from big data

Given current wariness in the usefulness of big data, it is necessary to clarify the technology’s current limitations, and identify possible approaches that enable the fulfillment of its potential going forward. The traditional paradigm of big data is deductive in nature. Specific questions (inputs) are asked, and discrete answers (outputs) are given—such as whether or not to order a specific diagnostic test. This is deemed “clinical decision support”: algorithm as hammer, and clinical problem as nail.

By intermediating the interaction of the data and the algorithm, clinician-investigators play a fundamental role here. As discussed, without appropriate intermediation, these predictions are subject to distortion resulting from inappropriate algorithm training. In addition, clinician-investigators impose rigor through a deliberate approach to data collection, to foster internal and external validity.^30^ They also impose structure through contextualization in the provision of care, to prevent the decoupling of predictions from clinical relevance. For example, Wells’ criteria for prediction of pulmonary embolism relies heavily on clinical discretion and is less accurate without it.^34^

Nonetheless, the potential of big data within this schema is limited, capable only of incremental improvements in patient care by offering a binary endorsement late in the decision-making process—such as for or against a CT scan. Furthermore, the generalizability of this approach has been brought into question, due to the impact of unseen data. For instance, when comparing outside hospital cases to cases used for training, the performance of deep learning models evaluating chest radiographs for the detection of pneumonia was significantly lower 60% of the time.^35^

## The new paradigm: inductive reasoning from big data

A future model for the use of big data is to harness its potential for inductive reasoning.^36^ In this model, few predictions enter, and many questions exit. This may be thought of as “clinical decision questioning”: conventional clinical practice as dented nail, and algorithm as claw. The new paradigm is accomplished by recognition, and illumination, of false positives and false negatives.

A famous nonphysician—Dr Seuss—once stated that “sometimes the questions are complicated, and the answers are simple.” The power of algorithms here is to liberate the human predictive process from preconceived lenses in data solicitation and/or interpretation.^37^ Inductive predictions can unshackle clinical decisions from the narrowness and biases inculcated by human medical training (and manifest in the clinical gestalt).

Inductive algorithms have already been employed to discover causal relationships in datasets with large amounts of unlabeled data. Genome sequences, pathology slides, and radiology images have all been leveraged by inductive algorithms to derive novel relationships undiscovered by human interpretation alone.^38–40^ It is likely not all derived relationships will be clinically impactful, as this approach also is susceptible to noncausal correlations. However, the hypothesis-generating capabilities of these methods have shown particular use for outputs with low prevalence, in which reductive thinking may be especially detrimental.^41^ Large, complex datasets with ever-smaller disease prevalence, progressing towards N-of-1: these are the exact parameters of personalized medicine.

Thus, an inductive approach offers revelation of formerly missing, impactful features while retiring preexisting, obsolete ones. It can drive inclusion of these new features in future datasets via refinement of existing measurement tools and additional of novel ones.^42^ Efficacy, representativeness and generalizability of research are all heightened in this schema. Inclusion of features less routinely considered in clinical care—through advancement of history-taking, diagnostic work-up, and treatment processes—may likewise improve outcomes.

For instance, information on social determinants of health (such as zip code, socioeconomic status, and educational background) has been demonstrated to improve prognostication and treatment planning for patients at risk for coronary heart disease compared with biomedical considerations alone.^43^ Used across clinical scenarios, inductive models could help providers prioritize evaluation and targeting of similarly under-investigated, high-impact features going forward.

Glymour et al. stated: “methodological innovation is not merely about applying novel methods to improve our estimation in the third decimal point. New data and new computing power should allow us to approach problems differently”.^20^ Early utilization of big data in an inductive manner can help redesign medical research and the clinical care emerging from it.

## Harmony of data, algorithms, and clinicians for personalized medicine

Big data’s potential for health is profound. At the preclinical stage, it can fill research voids (through trial emulation on preexisting datasets) and accelerate the movement of research from the bench to the bedside (through computational systems biology).^4,44^ At the clinical stage, it can better expound social determinants of health (by highlighting areas of disease uncertainty poorly explained by biology alone) and elucidate individual phenotypic nuances (by enabling multidimensional measurement of a given patient).^45^ In these ways, it offers the quickest route towards personalized medicine—through which health management is rigorously individualized.

Big data’s potential for care is also significant. Knowledge accumulation may not, in fact, be the physician’s greatest value to patients. Rather, physicians and patients alike flourish most when the “retention, access, and analysis” of knowledge by providers is delegated to algorithms, creating an opportunity for return to the “particularly human aspects of the profession”.^46^ Moreover, additional value is created through such human–computer partnership. Enhanced interaction empowers collection of those intimate data points solicited via a thoughtful history and a thorough physical.^47^ These are the very data points essential to bridge social circumstances with medical factors for optimization of care. The much feared elimination of humans from this scene is unlikely, as the last mile of big data (implementation of recommendations from prediction to action taken clinically) relies unequivocally on human–human contact.^17,47^ As such, big data offers an expedient return towards personified medicine—through which care is comprehensively humanistic.

However, the possible risks of big data—used deductively or inductively—arise from the inputs themselves. Optimal use of burgeoning technologies from newfound oceans of data requires stewardship of the data’s integrity.

**Fig. 1:**
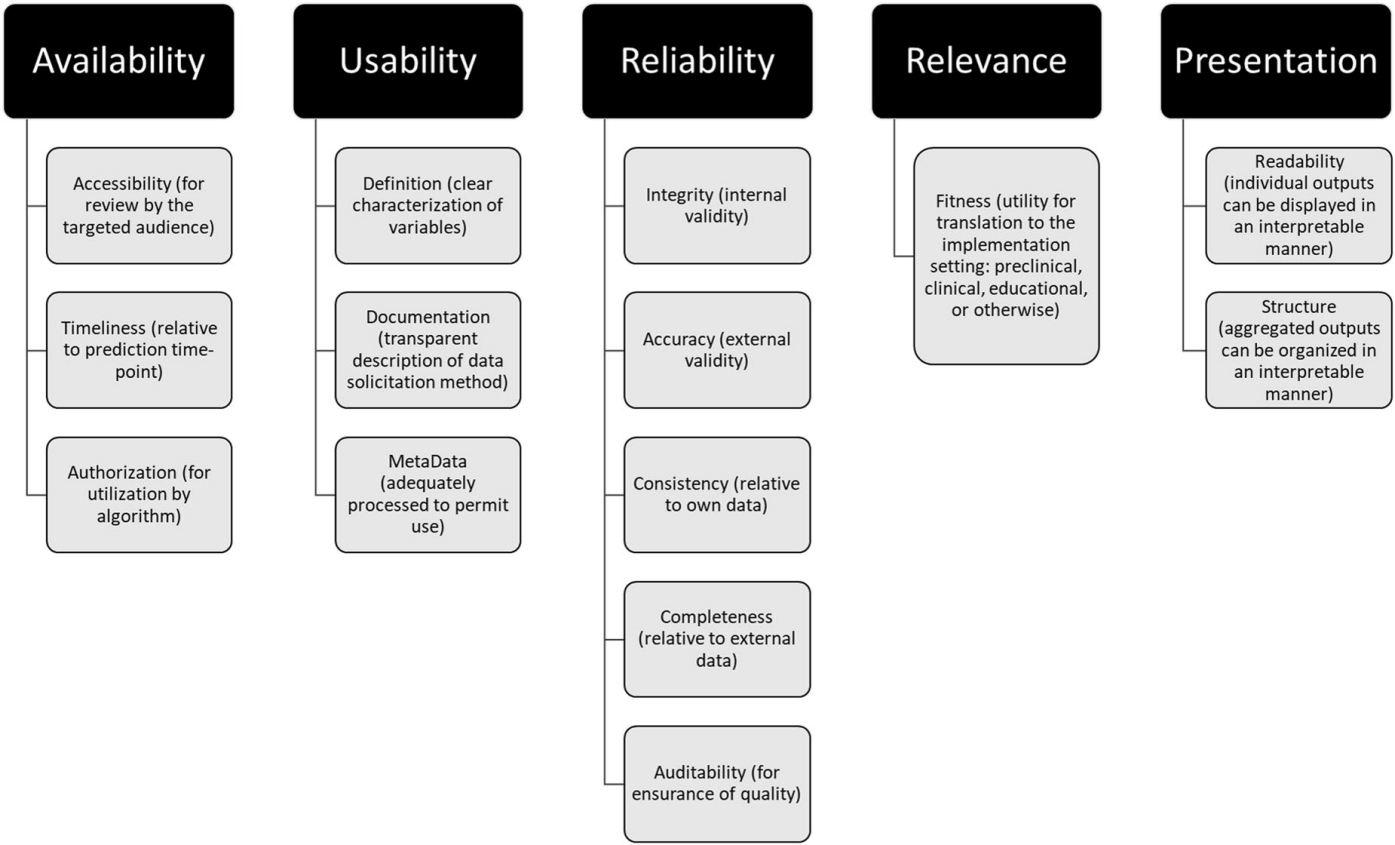
Guidelines describing quality standards for analytical datasets (used and modified with permission from Cai and Zhu^51^

Several strategies can support these goals. Annotation of training datasets with labeling metadata, by documenting biases intrinsic to them (such as sampling imbalance), can heighten transparency.^48^ In turn, redesign of methods for data collection (specifically relating to peripheral digital platforms) can ensure data variety beyond volume alone.^49^ For example, deliberate outreach efforts can be made by investigators to populations with poorer healthcare access. Imputation of heterogeneity to datasets and utilization of federated methods can support data veracity through inclusion of diverse feature sets when outreach efforts cannot be feasibly conducted.^50^ Dataset quality standards and minimum thresholds of inclusiveness used for analysis should likewise be adopted by journals to promote the utility of what they publish^51^ (Fig. 1). Finally, transparency into the characteristics of datasets should be provided to practitioners attempting to interpret emerging studies.

Mobilization of the technology itself in an inductive fashion can also support these appraisals. For example, methods like contrastive principal component analyses, which compare multidimensional enrichment patterns between datasets, are capable of visualizing ingrained data biases. Identification of the shortcomings of datasets offers one path to improving the utility of studies.^52^

Across all of these strategies, privacy of patient health information (PHI) must be prioritized. Increasing magnitude and dimensionality of data threatens to compromise patient anonymity even in de-identified databases.^53^ Compromise of privacy amidst accelerating data generation and use threatens the medical, financial, and social wellbeing of patients: for instance, discrimination in health insurance and job employment on the basis of PHI can perpetuate health disparities by impacting access to services and medications.^6,7^

As claimed by Confucius, “real knowledge is to know the extent of one’s ignorance.” To this end, awareness of data deficiencies, structures for data inclusiveness, strategies for data sanitation, and mechanisms for data correction can help realize the potential of big data for a personalized medicine era. Simultaneously, they can avoid risks of perpetuation of health inequity amidst widespread adoption of novel applications of big data.
